# Painful skin ulcers in psoriasis patients after taking methotrexate-containing traditional Chinese medicine: a retrospective case series

**DOI:** 10.3389/falgy.2025.1634055

**Published:** 2025-06-23

**Authors:** Yunhong Zheng, Yinzi Dubai, Ruizhe Wang, Suju Luo

**Affiliations:** ^1^ Department of Dermatovenereology, Tianjin Medical University General Hospital/Tianjin Institute of Sexually Transmitted Disease, Tianjin, China; ^2^ Department of Dermatology, Ulanqab Central Hospital, Inner Mongolia, Ulanqab, China; ^3^ Department of Dermatology, Gem Flower Xi'an Chang Qing Staff Hospital, Xi’an, China

**Keywords:** psoriasis, methotrexate toxicity, skin ulceration, traditional Chinese, immunosuppressive

## Abstract

**Background:**

Traditional Chinese Medicine (TCM) has demonstrated certain efficacy in the treatment of psoriasis, a chronic papulosquamous skin disease. However, in rare instances, the use of TCM may exacerbate cutaneous lesions. This study aims to explore whether the methotrexate (MTX) component in TCM may increase the risk of cutaneous ulceration in patients with psoriasis. MTX is also an immunosuppressive agent widely used in the treatment of various dermatologic conditions.

**Methods:**

We retrospectively analyzed the medical records of five patients who developed painful skin ulcers at primary psoriatic sites after taking TCM. Evaluated indicators included hemocytopenia, liver function, serum MTX concentration, and ABCB1 gene polymorphisms. Histopathological examination was also performed on ulcerative tissue samples.

**Results:**

Among the five patients, two developed hemocytopenia and two had abnormal liver function. Serum MTX concentrations in two patients ranged from 0.03 to 0.11 μmol/L (<0.1 μmol/L at 72 h after MTX administration). The ABCB1 genotypes AA and AG were detected in two different patients. Histopathological findings revealed dyskeratosis of keratinocytes, dermal vasodilation, and inflammatory cell infiltration. Rescue treatment with oral folic acid was administered to three patients, leading to complete healing of all lesions within two weeks. The remaining two patients showed gradual improvement in skin ulcers after discontinuing TCM.

**Conclusions:**

TCM containing MTX may induce skin ulceration in rare cases among patients with psoriasis.

## Introduction

Methotrexate (MTX) is an anti-folate drug possessing immunosuppressive, anti-proliferative, and anti-inflammatoryactivities (1). This drug has been used to treat many diseases including cancers, ectopic pregnancy, and rheumatoid arthritis (1–3). Psoriasis is a chronic, immune-mediated papulosquamous skin disease (4). MTX has a significant therapeutic effect on various types of psoriasis and is considered the first-line drug for systemic treatment of psoriasis (5, 6). The recommended maximum dose of MTX varies in different diseases. High-dose of MTX is used in patients with aggressive B-cell lymphoma (2), whereas low-dose of MTX is used to treat rheumatoid arthritis and psoriasis (7).

MTX can cause a variety of adverse effects, such as bone marrow suppression, hepatotoxicity, nephrotoxicity, and skin damage (8–10). Although relatively rare, several cases of cutaneous ulceration have been reported in psoriasis patients treated with low-dose MTX (11–13). Berna, R. et al. reviewed 114 cases of methotrexate-associated skin ulcers through the PubMed and Embase databases, of which 19 cases (16.7%) presented as localized ulcers (14). ABCB1 gene polymorphisms can influence MTX transport under different pathological conditions (15). In patients with psoriasis, ABCB1 gene polymorphisms play a significant role in the efficacy of MTX.

Traditional Chinese medicine (TCM) is an alternative and effective treatment option for patients with psoriasis (16, 17). However, there have also been individual cases in which cutaneous lesions worsened after taking TCM (18). The mechanisms underlying TCM-induced adverse skin reactions remain unclear. This study aims to investigate whether the MTX component contained in TCM may increase the risk of cutaneous ulceration in patients with psoriasis.

## Materials and methods

This study retrospectively analyzed the medical records of five psoriasis patients who presented with painful skin ulcers as the main manifestation after oral administration of TCM from April 2019 to February 2025. The inclusion criteria were as follows: a confirmed diagnosis of psoriasis; clinical presentation primarily characterized by painful skin ulcers; a clear history of compound TCM use, with either detectable MTX content in the compound TCM taken by the patient, or detectable serum MTX after TCM administration, or histopathological findings consistent with MTX-induced skin toxicity, or complete healing of skin ulcers within two weeks after folic acid supplementation; all enrolled patients signed written informed consent permitting the disclosure of their case-related information.

Laboratory evaluations included assessments of hemocytopenia, liver function, and serum MTX concentration. Two patients underwent ABCB1 gene polymorphism testing. The procedure was as follows: Genomic DNA was extracted from the peripheral blood of the subjects using the MagPure Buffy Coat DNA Midi KF Kit. The DNA was then randomly fragmented into segments primarily ranging from 100 to 500 base pairs using BGI's enzyme kit (Segmentase, BGI). Magnetic bead enrichment was performed to select DNA fragments between 280 and 320 base pairs. After end repair and A-tailing, T4 DNA ligase was used to ligate adapters containing barcode sequences. The ligated products were purified using Axygen beads, followed by LM-PCR amplification to enrich DNA fragments flanked by the adapters, thereby constructing individual DNA libraries for each patient. The libraries were hybridized and enriched using a capture chip. The enriched products were quantified with an Agilent 2,100 Bioanalyzer and BMG, then pooled in equimolar amounts. A total of 160 ng of the pooled DNA was used for single-strand separation and circularization, yielding single-stranded circular DNA libraries with one adapter. Finally, gene sequencing was performed using the MGISEQ-2000 platform developed by BGI. In addition, ulcer biopsies from two patients were subjected to histopathological analysis.

We quantified the methotrexate content in the compound capsules of traditional Chinese medicine (TCM) taken by the patients. The analysis was conducted using an ultra-performance liquid chromatography-tandem mass spectrometry (UPLC-MS/MS) system (Waters ACQUITY UPLC H-Class system coupled with a Waters Xevo TQ-S micro mass spectrometer), employing the MRM (multiple reaction monitoring) method for quantification. Detailed methodology: The TCM capsules were ground into a powder, and 0.5 g of the powder was weighed and mixed with 10 ml of methanol. The mixture underwent ultrasonic extraction for one hour, followed by centrifugation at 12,000 rpm for 10 min at 4°C. For liquid capsules, the supernatant was directly subjected to mass spectrometric analysis.

## Results

Tables 1, 2 list the main clinical findings observed in the five patients. In addition to the ulceration in primary psoriatic lesions, one case showed oral ulceration and two cases gastrointestinal discomfort (i.e., diarrhea and nausea). Laboratory examinations revealed two cases of hemocytopenia and two abnormal liver function. Two cases showed serum MTX concentration of 0.03–0.11 μmol/L (<0.1 mol/L 72 h after MTX intaking), while the remaining patients had undetectable levels, likely due to the prolonged interval since MTX discontinuation. Among the two patients tested for *ABCB1*polymorphisms, one patient had the *ABCB1*genotype AA and the other AG, the remaining three patients refused examination due to financial reasons. Analysis of ulcer biopsies showed dyskeratosis of keratinocytes, focal dermis-epidermal separation, dermal vasodilation, and infiltration of multiple inflammatory cells including neutrophils, eosinophils, and lymphocytes.

**Table 1:** Clinical manifestations of the five cases.

Case No.	Age, year/sex	Past history	Clinical manifestations			Name of Chinese herbal medicine taken	The dosage of MTX used	Source of MTX dosage selection	Time of ulcer onset after taking MTX
			Cutaneous involvement	Mucosal involvement	Others				
1	59/female	Vitiligo	Bright red erosive lesions on the lip mucosa; desquamative erythematous plaques with partial erosions on the lower back and buttocks	Oral ulcers	Diarrhea	Anti-Relapse No. 1 Capsule” (Manufacturer: Tangshan Jingdong Dermatology Research Institute)	15 mg/w	Self-doubled dosage	1 Week
2	61/male	Post-appendectomy	Generalized erythema with desquamation and scattered painful ulcers	–	–	No official name*	15 mg/w	Prescribed dosage	1 month
3	36/male	–	Scattered painful ulcers within plaques on the trunk and both lower limbs	–	Diarrhea	No official name*	15 mg/w	Prescribed dosage	1 month
4	68/male	Postoperative duodenal ulcer	Scattered ulcers within psoriatic plaques on the trunk and limbs.	–	–	No official name*	15 mg/w	Prescribed dosage	1 month
5	67/male	–	Large scaly erythematous plaques on the trunk and limbs, with multiple crusted ulcers on the plaques.	–	–	No official name*	15 mg/w	Prescribed dosage	1 month

* Because of China's cultural traditions of “experienced traditional Chinese medicine practitioners and folk remedies”, some folk remedies and privately formulated medicines lack official names and manufacturers.

**Table 2:** Laboratory analysis performed for the five cases.

Case No.		Biological data			Serum MTX level (u mol/L)	*ABCB1* genotype
	WBC (×10^−9^/L)	Hb (g/L)	PLT (×10^−9^/L)	Deranged liver function		
1	5.99	134	294	AST: 90 U/L ALT: 85 U/L	0.11	AA
2	8.93	110	552	Albumin: 21 g/L	Unmeasured	Unmeasured
3	4.47	130	57	N	0	Unmeasured
4	6.53	147	340	N	Unmeasured	Unmeasured
5	1.46	136	16	N	0.04	AG

MTX, methotrexate; N, normal range; Hb, serum hemoglobin level: Normal range 130–175 g/L; PLT, platelet count: Normal range 125–350 × 10^−9^/L; Albumin: Normal range 35–55 g/L; ALT, alanine aminotransferase: Normal range 8–40 U/L; AST, aspartate aminotransferase: Normal range 5–40 U/L; WBC, white blood cell: Normal range 4.0–10.0 × 10^−9^/L.

**Figure 1:**
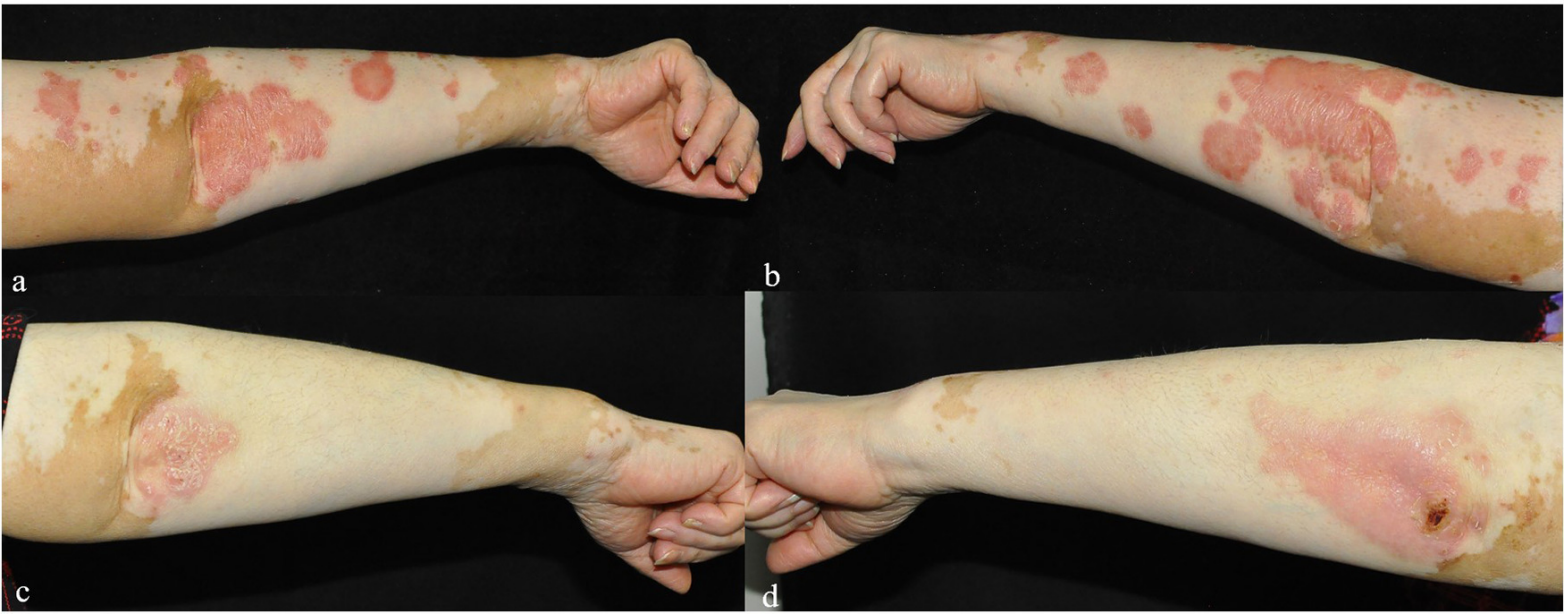
**(a–d)** The changes in the bilateral upper limb lesions in Case 1 before **(a,b)** and one week after folic acid supplementation **(c,d)**.

**Figure 2:**
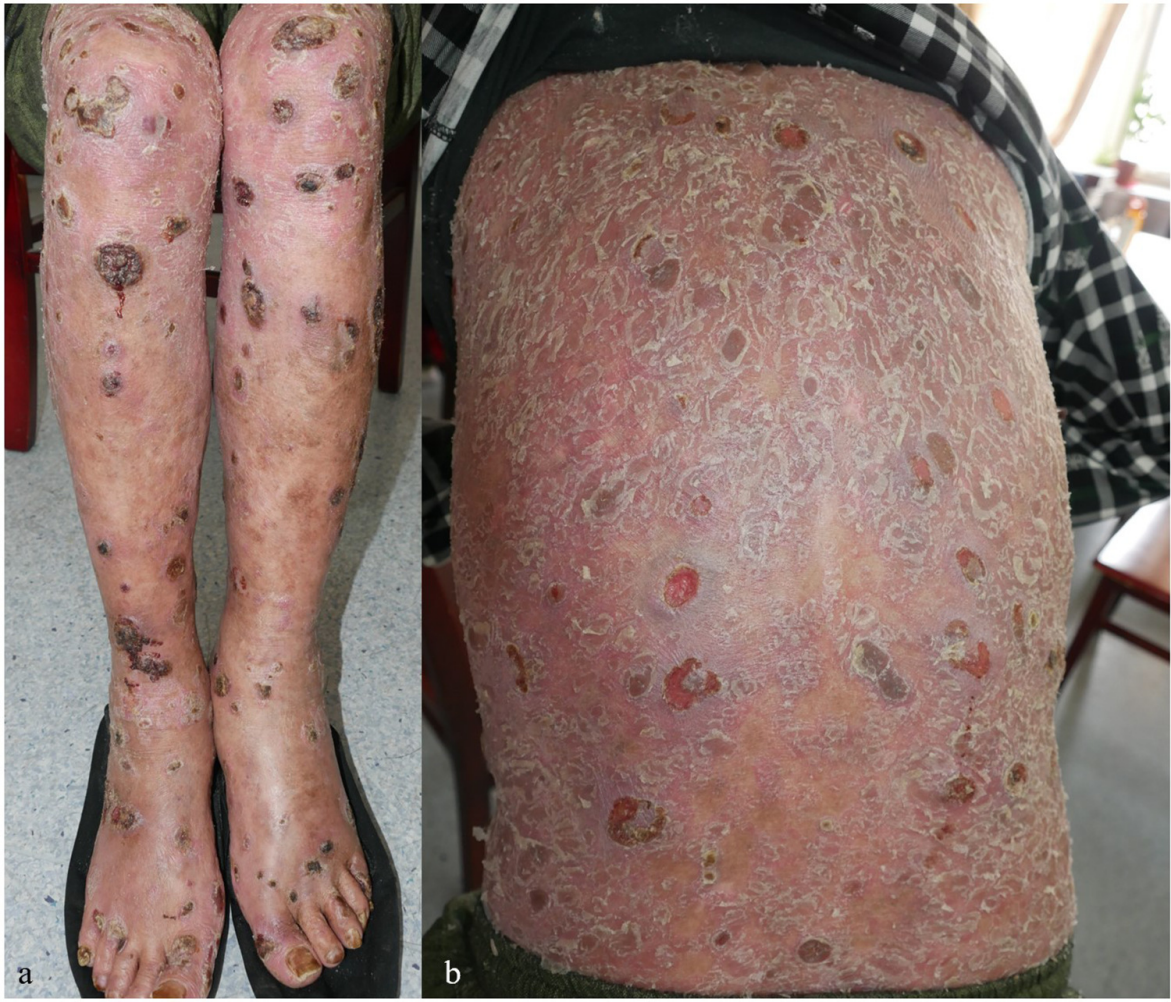
**(a,b)** Skin lesion characteristics of the lower limbs and back in Case 2 before treatment.

**Figure 3:**
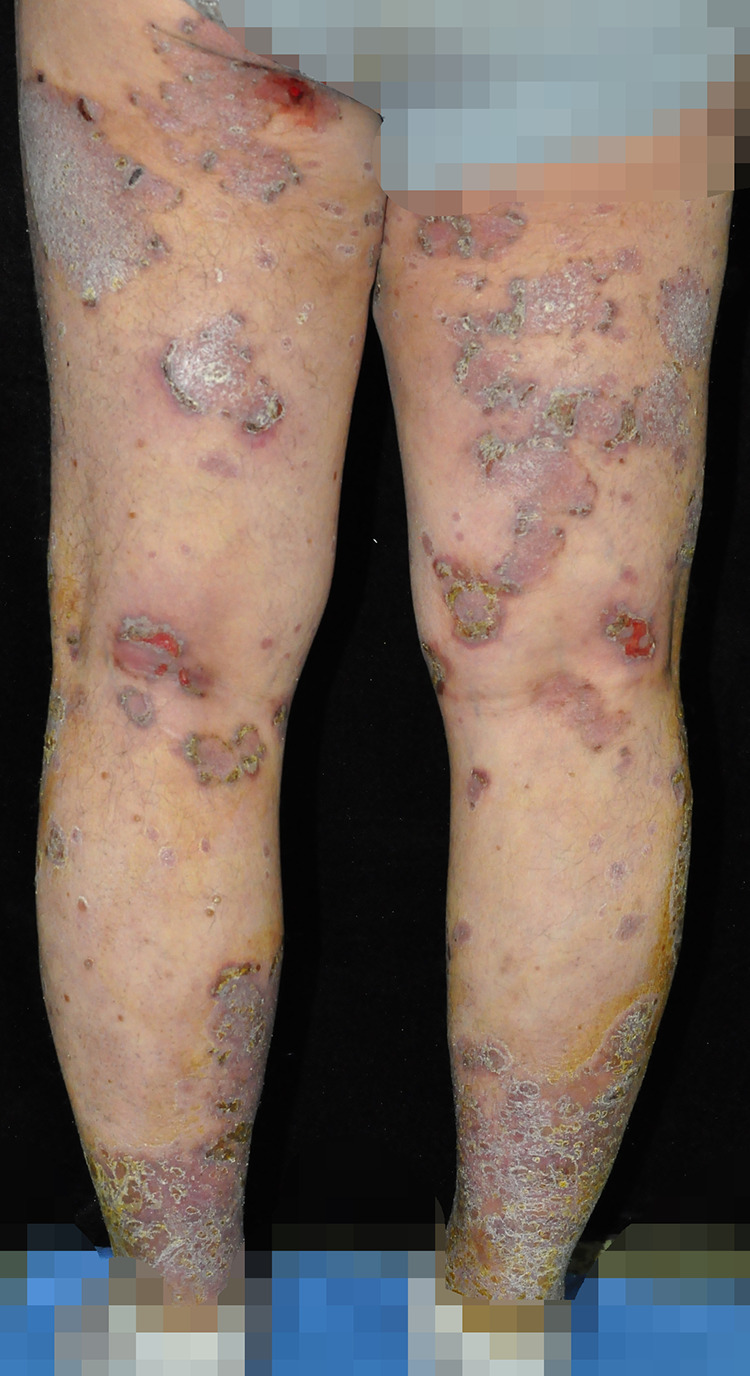
Skin lesion characteristics of Case 3 before treatment.

**Figure 4:**
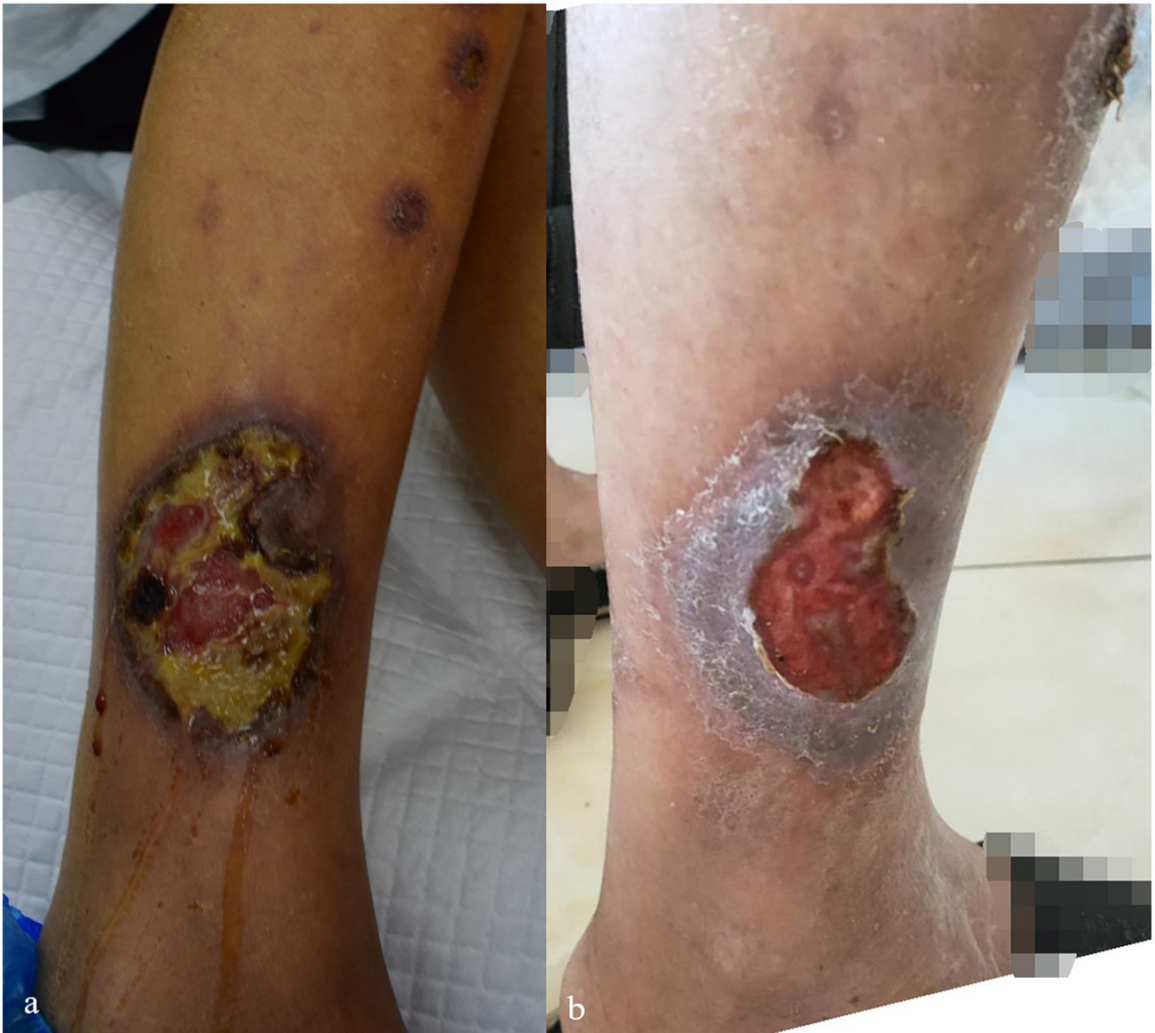
**(a,b)** Skin lesion characteristics of Case 4 before treatment and 3 weeks after discontinuing TCM compound capsules containing MTX.

**Figure 5:**
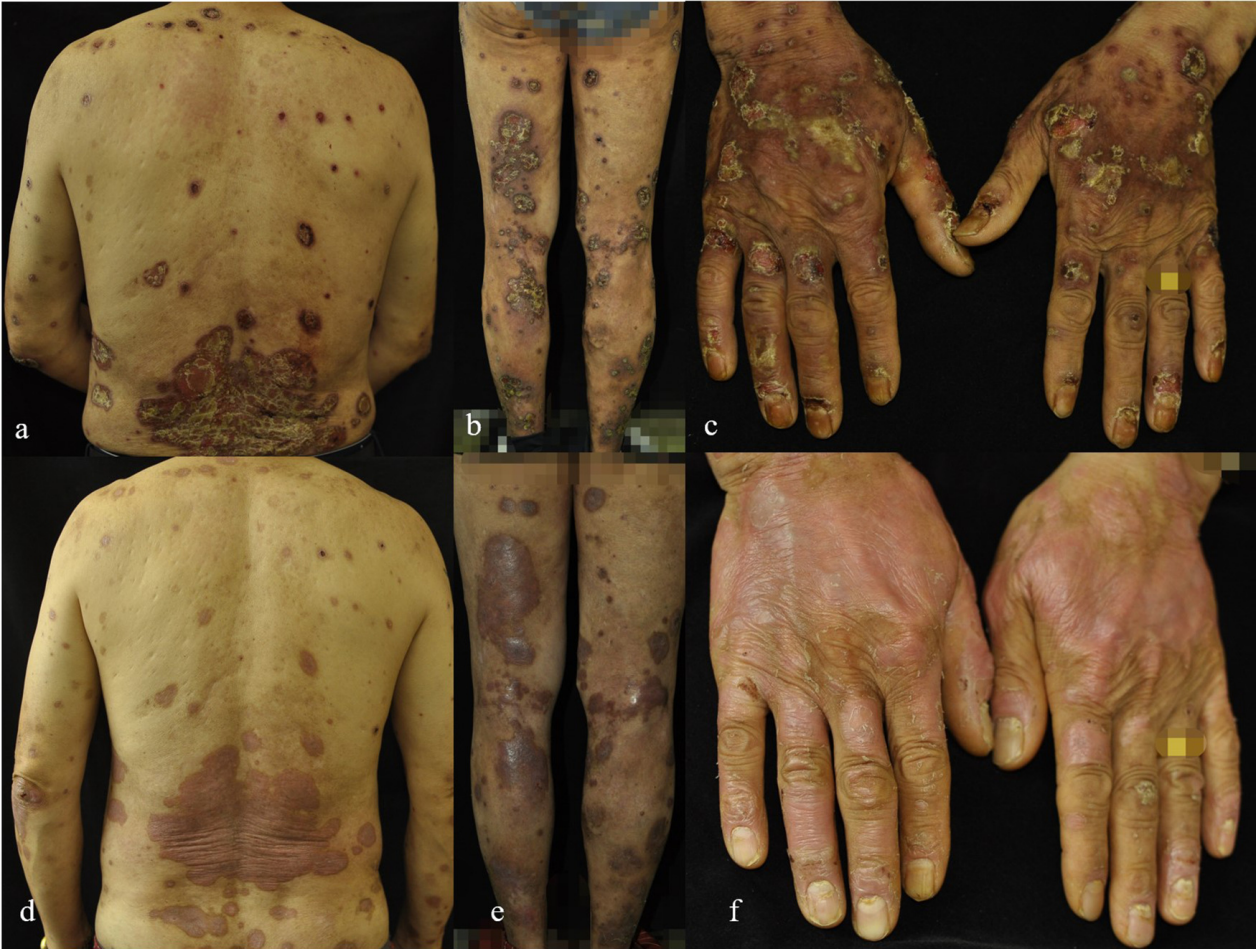
**(a–f)** The changes in the lesions on the back, flexor sides of both lower limbs, and dorsum of both hands in Case 5 before **(a–c)** and one week after folic acid supplementation treatment **(d–f)**.

**Figure 6:**
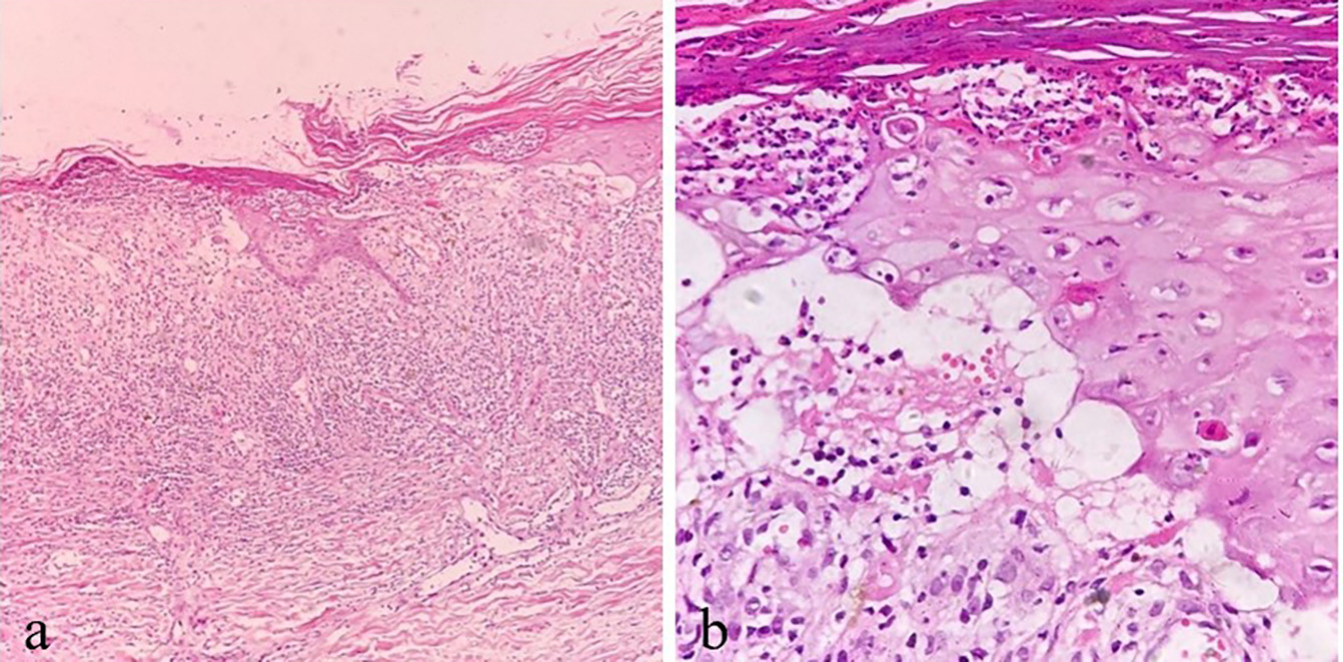
A(40×), b(100×): illustrative histopathological images of Case 4. **(a)** Thick parakeratosis, epidermal necrosis, ulcer formation, dermal vasodilation, and inflammatory cells in the dermis. **(b)** High-power view of parakeratosis with neutrophilic abscess, dyskeratosis of keratinocytes, focal dermis-epidermal separation, and scattered lymphocytes in the dermis.

Oral folic acid was given in three patients, resulting in complete healing of all lesions within two weeks (Table 3). The remaining two patients showed gradual improvement after discontinuing the TCM. Figures 1–5 shows the lesion changes in these five cases before and after treatment with folic acid. Figure 6 demonstrates the histopathological images for Case 4.

**Table 3:** Treatment and outcome of the five cases.

Case No.	Treatment		Time to skin resolution
	Drug discontinuation	Folic acid supplement	
1	TCM	10 mgTid	1 week
2	TCM	–	4 weeks
3	TCM	10 mgTid	2 weeks
4	TCM	–	4 weeks
5	TCM	10 mg Tid	1 week

## Discussion

This study reports five cases of psoriasis patients who developed skin ulcers after taking MTX-containing TCM. MTX is a cytotoxic drug structurally similar to folic acid. After entering cells via the reduced folate carrier (RFC-1), MTX irreversibly binds to the enzyme dihydrofolate reductase (DHFR), preventing the formation of tetrahydrofolate (19). As a result, it competitively inhibits the S phase of cell division and suppresses cell proliferation, thus preferentially affecting actively proliferating cells (20). In psoriasis, proliferating lymphocytes and epithelial cells may become targets of MTX (19). Folate deficiency within psoriatic lesions may lead to the selective accumulation of MTX, thereby enhancing its cytotoxic effects (21). MTX-induced skin ulcers in psoriasis can be classified into two types (22): type I ulcers occur on psoriatic plaques, while type II ulcers appear outside of psoriatic lesions. Type I ulcers are generally shallower than type II ulcers. In this study, all five patients exhibited type I ulcers after oral administration of TCM.

MTX toxicity is influenced by multiple factors including drug dose, renal insufficiency, advanced age, folate level, serum albumin reduction, and gene polymorphism (10, 11, 23). As the kidney is responsible for the elimination of MTX, decreased renal function with age contributes to MTX toxicity (23). In this study, four of the five patients were over 55 years old. Therefore, MTX toxicity tends to occur in older patients. Two cases showed serum MTX concentration of 0.03–0.11 μmol/L (<0.1 mol/L 72 h after MTX intaking), suggesting that MTX overdose may also account for the painful skin ulcers observed. However, MTX dermal toxicity was still observed in 1 case who had an undetectable level of serum MTX. Therefore, serum MTX concentration may not be a reliable predictor for the MTX toxicity.

TCM-related skin ulceration is rarely encountered in psoriasis patients. Genetic background of patients may be an important determinant of TCM-related adverse reactions. Compelling evidence indicates that the transporter *ABCB1*polymorphisms affect the transport of MTX in different pathological contexts (24–26). The P-glycoprotein (P-gp) encoded by the ABCB1 gene is an important transmembrane transporter located on the cell membrane, which can influence the transmembrane transport, tissue distribution, and clearance rate of various drugs, including methotrexate (MTX) (24). In patients with psoriasis, ABCB1 gene polymorphisms have a significant impact on the therapeutic efficacy of MTX. Patients with certain genotypes (such as AA and AG) tend to have lower expression levels of P-gp, leading to increased accumulation of MTX in tissues, which in turn exacerbates drug toxicity, including severe adverse effects such as skin ulceration. In contrast, patients with the GG genotype generally exhibit higher P-gp expression, resulting in enhanced drug efflux, lower MTX levels in the body, and consequently a reduced risk of adverse reactions (27). Our results suggest that the deleterious effects incurred by MTX-containing TCM are also affected by the *ABCB1* gene polymorphisms. Among the two patients tested for *ABCB1* polymorphisms, one was the AA genotype, and the other AG genotype. The *ABCB1* AA or AG genotype seems to be more susceptible than the GG genotype to MTX toxicity. Therefore, when using MTX-containing traditional Chinese medicine in patients with psoriasis, the polymorphisms of the ABCB1 gene may need to be taken into consideration. Detection of ABCB1 gene polymorphisms holds promise for guiding individualized MTX therapy in clinical practice. This is especially important for patients using MTX-containing traditional Chinese medicine, as dosage and monitoring strategies can be tailored according to the patient's genotype, thereby reducing the risk of adverse reactions and improving treatment safety.

TCM is composed of a large number of bioactive compounds. Although our data indicate the increased risk of dermal toxicity by MTX, we cannot exclude the possibility that other chemical agents from TCM may also contribute to skin ulceration observed in psoriasis patients. Nevertheless, discontinuation of TCM or supplementation of folic acid is useful in resolving the TCM-related skin ulcers. The limitations of this study include a small sample size and the lack of comprehensive genetic data. In the future, it will be necessary to expand the sample size and incorporate a broader range of genetic backgrounds and multicenter data to further investigate the predictive and interventional significance of ABCB1 gene polymorphisms in MTX-induced cutaneous toxicity.

## Conclusion

In summary, our work demonstrates that MTX-containing TCM play a role in skin ulceration in rare cases of psoriasis patients. Understanding the mechanism for TCM-induced dermal toxicity is important to improve the safety of TCM-based therapy against psoriasis.

## Data Availability

The raw data supporting the conclusions of this article will be made available by the authors, without undue reservation, to any qualified researcher.
